# Analyses of haplotypes of *TLR2* and *TLR3* genes for COVID-19 prognosis in a cohort of professionals who worked in the first pandemic wave in Belém-PA, Brazil

**DOI:** 10.3389/fgene.2025.1659269

**Published:** 2025-10-02

**Authors:** Marcos Jessé Abrahão Silva, Luiza Raquel Tapajós Figueira, Daniele Melo Sardinha, Eliete Costa da Cruz, Natasha Cristina Oliveira Andrade, Sebastião Kauã De Sousa Bispo, Thiago Augusto Ferreira Dos Anjos, Everaldina Cordeiro Dos Santos, Ana Judith Pires Garcia, Luana Nepomuceno Gondim Costa Lima

**Affiliations:** ^1^ Postgraduate Program in Parasitic Biology in The Amazon (PPGBPA), State University of Pará (UEPA), Belém, Brazil; ^2^ Nursing Department, University of the Amazon (UNAMA), Ananindeua, Brazil; ^3^ Postgraduate Program in Epidemiology and Health Surveillance (PPGEVS), Evandro Chagas Institute (IEC), Ananindeua, Brazil; ^4^ Bacteriology and Mycology Section, Evandro Chagas Institute (IEC), Ananindeua, Brazil

**Keywords:** COVID-19, TLR2, TLR3, Brazil, single-nucleotide polymorphism

## Abstract

Coronavirus disease 2019 (COVID-19) is a multisystemic disease caused by SARS-CoV-2 that can lead to several pulmonary illnesses according to the immunological contexts of the individual. Haplotypes consist of single-nucleotide polymorphisms (SNPs) within candidate genes for diseases. *TLR2* and *TLR3* are genes located on human chromosome 4 (chr:4) and composite a haplotype that influence immune signaling and inflammatory pathways. The purpose of this article was to genetically analyze *in silico* a cohort of professionals from Belém-PA during the first wave of the pandemic using SNPs rs3804100, rs3775290, and rs3775291 on the human chr:4. This is a computational genomic design using bioinformatic software and machine-learning technologies on epidemiological data of Sanger sequencing data. Regarding the findings, none of the alleles formed by the haplotype showed statistical significance for symptomatology or disease severity. The haplotype block was not significant between the SNPs analyzed despite a high permutation rate of alleles at the beginning of the variance of the individual genomic data. Then, the *TLR2–TLR3* haplotype (SNPs rs3804100, rs3775290, and rs3775291) showed little determination in the clinic of individuals with COVID-19 in Belém (Northern Brazil), which may indicate differences in collective genetic patterns and/or epigenetic influences compared to other more affected populations that have the same haplotype pattern.

## 1 Introduction

Coronavirus disease 2019 (COVID-19) was declared a pandemic by the World Health Organization (WHO) in March 2020 due to its high spread. The epidemic came on abruptly and left the Public Health System under severe pressure and not knowing how to manage the cases that arose, inducing infected people into a critical state and consequently many deaths (Chen et al., 2020; Choudhury and Mukherjee, 2020). As it is a highly transmissible pathogen, it was recommended that people should be restricted from circulating to reduce contamination rates (Bastos et al., 2021). However, health professionals did exactly the opposite of what was recommended for most of the population, which made them more susceptible to acquiring the disease (Coelho et al., 2022).

There are different clinical manifestations, depending on the host’s immune status, the genetic variability of the virus circulating at the time, and other genetic and even sociodemographic aspects; in this sense, the symptomatology of COVID-19 ranges from asymptomatic or with mild manifestations similar to other severe acute respiratory syndromes (SARS)—even acute respiratory distress, sepsis, septic shock, acute thrombosis, and/or multiple organ dysfunction. These complications often require hospitalizations for several days in ICUs, with many patients not surviving, and those who recovered would have to live with the sequelae left behind (post-COVID-19) (Cham et al., 2021; Fronteira et al., 2024).

The COVID-19 pandemic has affected the world in a big way, with the first pandemic wave characterized by rapid spread and high mortality. According to studies, from 1 January 2020 to 31 December 2021, there were approximately 5.94 million deaths worldwide. In Brazil, the first wave hit the country hard, showing the weaknesses of the health system and placing health professionals on the front line. Professionals faced long working hours, a lack of personal and collective protective equipment (PPE and CPE), and the anguish of dealing with a high number of critically ill patients. The emotional and physical impacts were significant, with many professionals suffering from anxiety and other mental health problems (Agrawal et al., 2021). According to the Brazilian Institute of Geography and Statistics (IBGE), approximately 1.5 million deaths occurred in 2020 in Brazil because of COVID-19. The front line of the pandemic in Brazil was marked by the dedication of health professionals, who fought to save lives in the midst of a troubled scenario (Almeida et al., 2020; COVID-19 Excess Mortality Collaborators, 2022).

Immunology explores the complex defense system of the body against pathogens. Innate immunity, the first line of defense, uses Toll-like receptors (TLRs) to recognize molecular patterns associated with pathogens (PAMPs). In particular, *TLR2* detects lipopeptides and bacterial peptidoglycans, whereas *TLR3* recognizes viral double-stranded RNA. Both TLRs play a crucial role in the detection of viruses, including SARS-CoV-2. Studies indicate that SARS-CoV-2 can be recognized using TLRs, including *TLR2* and *TLR3*, through different mechanisms, with the interaction of viral proteins with these receptors, leading to the activation of the innate immune response, thus preventing excessive inflammatory response (Dhangadamajhi and Rout, 2021; Choudhary et al., 2022; Machado et al., 2023).

Haplotypes are combinations of alleles at different markers along the same chromosome that are inherited as a unit. In research, their main purpose is to identify the causes of diseases, marking recombination events in family and population-based studies, and clinically, they apply to the greater or lesser correlation in predicting the severity of diseases. Single-nucleotide polymorphisms (SNPs) can originate from point mutations in DNA, which are intragenic and cause important biological effects such as association with complex diseases and reactions in responses to treatments in humans (Bafica et al., 2005; Chen et al., 2017; Bortolotti et al., 2021).

This study of the *TLR2*–*TLR3* haplotype, which includes two SNPs (rs3804100 in *TLR2* and rs3775290 and rs3775291 in *TLR3*), may aid in the identification of genetic differences that influence COVID-19 susceptibility and clinical progression, hence facilitating the development of individualized disease containment and treatment methods. These SNPs (rs3804100 in *TLR2* and rs3775290 and rs3775291 in *TLR3*) were selected because they are in nearby functional regions, previously associated in the literature with viral immune response and respiratory diseases (such as tuberculosis, influenza, and other viral infections) (Bucciol et al., 2022; Córdova-Dávalos et al., 2022; Chen et al., 2023; Silva et al., 2023a). Thus, investigating the haplotypes of SNP rs3804100 in the *TLR2* gene and SNPs rs3775290 and rs3775291 in the *TLR3* gene is a viable approach to understanding the immunogenetic mechanisms driving COVID-19 and its clinical symptoms (Chen et al., 2011; Vega et al., 2017; Sharif et al., 2020; Djordjevic et al., 2022).

Considering the impact of the pandemic on the workforce of healthcare professionals and the need to identify the association of haplotypes for the prognosis of COVID-19 in these professionals (Zangoue et al., 2021), along with the relevance of immunogenetics in this context, in this study, we aimed to analyze the haplotypes of the *TLR2* and *TLR3* genes on the human chromosome, in the genomic area near SNP rs3804100 of *TLR2* and SNPs rs3775290 and rs3775291 of *TLR3* in the labor cohort of Belém-PA, Brazil, from the first pandemic wave.

## 2 Materials and methods

### 2.1 Study design and ethical considerations

This study is characterized as a case–control type, with convenience sampling, and it is part of a larger scientific project. It followed the recommendations of the Strengthening the Reporting of Observational Studies in Epidemiology (STROBE) (Von Elm et al., 2014). This study protocol was approved by local ethics committees, and all subjects gave their written informed consent (Term of Free and Informed Consent—TCLE). This work was approved by the Research Ethics Committee of the State University of Pará—UEPA (CAAE: 38113620.1.0000.5174), with opinion number: 6.124.862. This research was carried out in accordance with the Helsinki Declaration (Helsinki Declaration, 1964) and Resolution No. 466/2012 of the Brazilian National Health Council (Brasil et al., 2012).

### 2.2 Settings and participants

This study was carried out in 10 health centers and institutions located in the city of Belém-PA, Brazil (in the Amazon Region—Northern Brazil). During the initial wave of the pandemic, individuals infected with COVID-19 were admitted to all collaborating institutions in Brazil (López-Juárez et al., 2021). Based on the acquisition of contacts that were appropriate for this research group, these institutions were selected at random. Epidemiological analyses were carried out using a questionnaire applied in person at health centers and analyses of genetic data sequenced from blood biological samples using molecular biology methods.

Professionals (in health, administrative, and general service areas) who actively worked during the first wave of the pandemic in the period between 1 April 2021 and 30 June 2020 (Bastos et al., 2021), who were directly and daily exposed to and infected by the virus in Belém, and who agreed to participate in the research by signing the consent form were included in this study. This cohort of professionals (convenience sampling methodology, institutions, and health centers selected) has already been portrayed by our research group in earlier studies involving other epidemiological and genetic analyses (Silva et al., 2023c; Silva et al., 2023b; Marcelino et al., 2025). All employees at healthcare facilities during this time may have been exposed to SARS-CoV-2. Because safety regulations were still developing in Brazil, medical facilities were overcrowded, masks were scarce, and N95 masks were not worn (Quigley et al., 2021).

### 2.3 Division of groups, variables, participants, sampling, and laboratory procedures

The study population questionnaires were evaluated to collect epidemiological data and potential association with genetic information of the participants, which were evidenced in demographic variables, comorbidities, signs and symptoms, professional category, and records of illnesses of intra- and extra-household contacts of the consanguineous and non-consanguineous types. Data included signs and symptoms associated with the first wave period, sex, age, employment, and comorbidities. Based on the patient’s self-report during the questionnaire, chest computed tomographic (CCT) data were included.

Sample collection was carried out between 1 June 2021 and 30 March 2022. Laboratory procedures were conducted in the Molecular Biology Laboratory—LABIMOL, Bacteriology and Mycology Section (SABMI) of the Evandro Chagas Institute (IEC). DNA extractions were performed using the DNeasy Blood and Tissue Kit (QIAGEN^®^, Venlo, Netherlands), following the manufacturer’s instructions. For all these professionals, SNP rs3804100 (T>C) of *TLR2* and SNPs rs3775291 (C/T) and rs3775290 (C/T) of *TLR3* were evaluated to correlate with the susceptibility and severity of COVID-19. The information on the SNPs (SNP ID) of *TLR2* and *TLR3* was retrieved from the National Center for Biotechnology Information—NCBI, dbSNP (http://www.ncbi.nlm.nih.gov/snp/; accessed on 1 January 2022) (Sayers et al., 2023). Between 1 November 2021 and 1 November 2022, the sample collections and questionnaires were performed. Polymerase chain reaction (PCR) was performed with Platinum Taq DNA Polymerase, DNA-free (Invitrogen^®^, Thermo Fisher Scientific Corporation, Waltham, Massachusetts, United States) using the thermocycler. The amplified products of PCR were subjected to electrophoresis in a 2% agarose gel containing 3.0 µL of Sybr Safe (Invitrogen^®^, Thermo Fisher Scientific Corporation, Waltham, MA, United States) to visualize the amplified DNA fragments in a photodocumenting device. *TLR2* SNP rs3804100 and *TLR3* SNPs rs3775291 and rs3775290 were selected together for the analysis of the present article due to their proximity in the genomic region of the same human chromosome.

The EasyPure PCR Purification Kit (TransGen Biotech Co.^®^, Beijing, China) was used to purify the PCR products in accordance with the manufacturer’s instructions. The BigDye X-Terminator Kit instructions were followed to submit the previously purified amplified products to the ABI 3130 Genetic Analyzer sequencer (Applied Biosystems^®^, Life Technologies, Thermo Fisher Scientific Corporation, Waltham, MA, United States) for the sequencing reaction. The Bioedit program version 7.2.5 was used to visualize and analyze the SNP areas of interest, and BLAST was then performed on the NCBI website (https://blast.ncbi.nlm.nih.gov/Blast.cgi/), obtained on 15 August 2023 (Hall, 1999).

The criteria and forms of grouping based on symptoms and severity of COVID-19 [AS, asymptomatic group—n = 91; SI, symptomatic group—n = 121 (divided into SCP, symptomatic with pulmonary involvement, with n = 34, and SSP, symptomatic without pulmonary involvement, with n = 5, based on the participants’ CCT data)], criteria for tomographic data evaluation of severity of COVID-19 through CCT (≥10% in CCT data, according to the guidelines provided by the WHO and the Ministry of Health) (Choi et al., 2020; UNA-SUS, 2020), sample size (N = 212) and power of sampling (p = 0.94), variables analyzed using the questionnaires, method of defining COVID-19 cases during the first wave in Brazil (based on Brazilian Ministry of Health recommendations), inclusion and exclusion criteria for participants, epidemiological data from the cohort, blood collection method, method of extracting DNA from samples, laboratory procedures, primers used, PCR and Sanger sequencing methods, and statistical investigation of agreement of each SNP with the Hardy–Weinberg equilibrium (HWE, p > 0.05) and comparative and associative analyses of genotyping results in a genotypic and allelic form of SNPs rs3804100, rs3775291, and rs3775290 with COVID-19 in this population evaluated (individually for each SNP) have already been reported in our previous studies, however, without the haplotypic investigation of this present work (Silva et al., 2023c; Silva et al., 2023b; Marcelino et al., 2025).

### 2.4 Bioinformatic and statistical analyses

Only subjects who had all SNPs correctly genotyped were included in the haplotype analysis, which was constructed from genotype data using the computer tool Haploview v4.2. At p < 0.05, statistical significance was established. Furthermore, the Haploview 4.2 tool was used to perform linkage disequilibrium (LD) blocks and haplotype association risk studies (Barrett et al., 2005). Thesias v3.1 software, which is based on the stochastic expectation–maximization technique for haplotype analysis, was used to estimate haplotype frequencies, HWE concordance, and their impacts. G*Power v3.1.9.7 software was used to measure sample size power using a Chi-square goodness-of-fit test (Kang, 2021). The power of the sample size, the normality of the variables, the Hardy–Weinberg equilibrium, and the epidemiological characteristics of the individuals in this cohort of professionals from Belém have already been established and calculated by previous studies carried out by our research group (Silva et al., 2023c; Silva et al., 2023b).

The level of connection between the examined haplotypes and COVID-19 (symptomatology, As and SI; and severity, AS, SCP, and SSP) was measured using the odds ratio (OR) and its 95% confidence interval (CI). The impact of each haplotype on COVID-19 is shown using the Thesias program as predicted phenotypic averages for a single dosage of each haplotype, together with the associated 95% CI in relation to the reference haplotype. The most prevalent haplotype in the study groups is the reference haplotype, which was determined using the Thesias program (Djordjevic et al., 2022). The statistical software program SPSS v26 (IBM Corp.^®^, Armonk, NY, United States) was used for all statistical analyses. The 95% CIs and ORs show the relative risk.

To contextualize the allele frequencies found in our local cohort within the spectrum of global genetic diversity, an *in silico* comparative analysis was performed using large-scale public data. The allele frequencies of SNPs rs3804100, rs3775290, and rs3775291, obtained through Sanger sequencing of our sample, were compared with the frequencies reported for the same variants in the Genome Aggregation Database (gnomAD), version 4.0.0. In particular, data from our Belém population were contrasted with the frequencies of the global aggregate and the “Latino/Admixed American” superpopulation available in gnomAD. The gnomAD database was accessed on 23 August 2025, and frequencies were extracted directly from the specific entries for each SNP in the portal. This analysis allowed us to assess how representative or divergent the genetic frequencies of our Amazonian cohort are in relation to broader reference populations (Gudmundsson et al., 2022).

## 3 Results

### 3.1 COVID-19 symptomatology parameter and haplotype analyses for this cohort

The chi-square goodness-of-fit test was used to calculate sample size power for the cohort population of symptomatic (N = 121) and asymptomatic (N = 91) COVID-19 individuals, with an error probability α of 0.05 and an effect size of 0.3. The actual real power (1-β error probability) is 0.94, which is higher than 0.80, and this is statistically acceptable. The results of the data sequenced and then analyzed revealed a pattern of allele frequency and haplotypic background without statistical significance in the influence of the investigated phenotype of COVID-19 symptomatology (Tables 1, 2).

**TABLE 1:** Allelic distribution and associative comparison of frequencies in the haplotypes for these groups.

Allele	Position	Minor allele frequency (MAF)	Frequency		HWE (p-value)	Association parameter (AS × SI)	AS × SI (*p*-value)	AS × SI [OR (95% CI)]
			(AS; n = 91)	(SI; n = 121)				
SNP rs3804100 (T>C)	−450	C (0.047)	95.28%	4.71%	0.47	Haplotypic background–CC	0.61	0.74 [0.23–2.34]
						Haplotypic background–CT	0.30	2.93 [0.37–22.94]
SNP rs3775291 (C>T)	−412	T (0.007)	99.29	0.70%	0.91	Haplotypic background–TC	0.71	1.58 [0.13–17.92]
SNP rs3775290 (C>T)	−459	T (0.25)	74.52%	25.47%	0.41	Haplotypic background–TC	0.79	1.06 [0.67–1.66]
						Haplotypic background–CC	0.21	4.20 [0.43–40.59]

**TABLE 2:** Distributive and association analyses of each haplotype in the evaluated groups to COVID-19, regarding the reference haplotype.

Haplotype	Frequency			AS × SI (p-value)	AS × SI (OR [95% CI])	R^2^ information
	Total (N = 212)	AS (n = 91)	SI (n = 121)			
Reference haplotype: TCC	70.51%	70.02%	70.71%	—	—	R^2^ = 0.97
TCT	24.09%	24.18%	23.86%	0.79	1.06 [0.67–1.66]	R^2^ = 0.96
TTC	0.50%	0.82%	0.70%	0.71	1.58 [0.13–17.92]	R^2^ = 0.69
CCC	3.1%	2.70%	3.1%	0.61	0.74 [0.23–2.34]	R^2^ = 0.76
CCT	1.6%	2.25%	1.60%	0.26	3.11 [0.42–22.83]	R^2^ = 0.64

**FIGURE 1:**
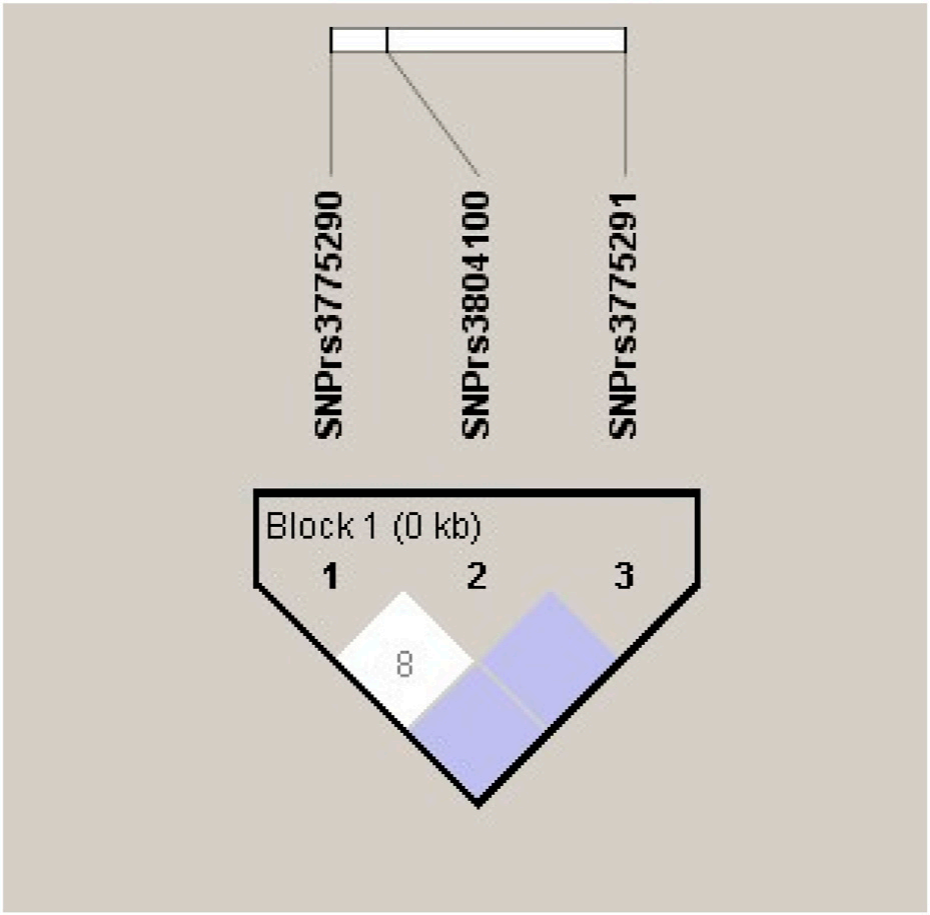
Linkage disequilibrium (LD) blocks and R^2^ values between the analyzed polymorphisms in the *TLR2* (rs3804100) and *TLR3* (rs3775290 and rs3775291) genes for the total cohort sample (N = 212).

To further examine the association of the candidate SNPs between the AS × SI groups, we estimated the LD and haplotype blocks; SNPs rs3775290 and rs3804100, SNPs rs3804100 and rs3775291, and SNPs rs3775290 and rs3775291 were in low LD with each other (r^2^ = 0.08, r^2^ < 0.08, and r^2^ < 0.08), showing low r^2^ values, indicating low co-inheritance between the variants in the studied group and their limited impact on the haplotype (Figure 1).

**FIGURE 2:**
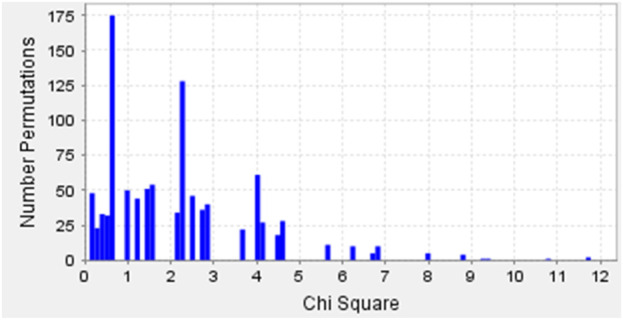
Permutation test results of SNPs rs3804100 (*TLR2*), and rs3775290 and rs3775291 (*TLR3*) showing Chi-square distributions for COVID-19 symptomatology in the Belém cohort.

Permutation tests were performed using Haploview software, running 1,000 iterations for individual SNPs to analyze the possible change in the phenotype of the symptoms related to the changes in the alleles (Table 3), and this statistical profile of permutations is shown in Figure 2.

**TABLE 3:** Results of significance tests for 1,000 permutations of SNPs regarding the change in the symptomatology phenotype of COVID-19 in this cohort (N = 212).

SNP ID	Chi-square value	Permutation *p*-value
rs3804100	0.073	1.0
rs3775291	0.113	1.0
rs3775290	0.116	0.99

### 3.2 COVID-19 severity parameter and haplotype analyses for this cohort

*In silico* analysis for the paired evaluation of the SNPs of this haplotype was carried out on this cohort of professionals evaluated in relation to the severity of the disease (Table 4).

**TABLE 4:** Comparative analysis of the association of each haplotype with the profiles of COVID-19 severity groups (regarding reference haplotype: TCC).

Haplotype	*p*-value	OR [95% CI]
AS (n = 91) × SCP (n = 34)		
TCT	0.82	1.07 [0.55–2.09]
TTC	—	1.0 [1.00–1.00]
CCC	0.91	1.09 [0.22–5.28]
CCT	0.40	2.59 [0.27–24.23]
AS (n= 91) × SSP (n= 8)		
TCT	0.22	1.97 [0.65–5.99]
TTC	—	1.0 [1.00–1.00]
CCC	—	1.0 [1.00–1.00]
CCT	—	1.0 [1.00–1.00]
SCP (n= 34) × SSP (n= 8)		
TCT	0.30	1.86 [0.56–6.36]
TTC	—	1.0 [1.00–1.00]
CCC	—	1.0 [1.00–1.00]
CCT	—	1.0 [1.00–1.00]

### 3.3 Comparative *in silico* analysis of SNP allele frequencies

To contextualize our findings within the global genetic diversity, we compared the minor allele frequencies (MAFs) of the studied SNPs in our cohort (Belém) with those from the Genome Aggregation Database (gnomAD v4.0.0) (Table 5). As shown in Table 5, the allele frequencies in our population markedly differ from global aggregates, particularly for rs3804100 (MAFBelém: 4.7% vs MAFgnomAD Global: 24.5%). Notably, our cohort’s frequencies are more similar, yet still they are distinct from those of the gnomAD Latino/admixed American superpopulation (e.g., rs3804100 MAF: 4.7% vs 10.2%).

**TABLE 5:** Comparative minor allele frequency (MAF) analysis of SNPs rs3804100, rs3775291, and rs3775290 between the Belém cohort (this study) and the gnomAD v4.0.0 database.

SNP	Allele (major > minor)	MAF–Belém cohort (N = 212)	MAF–gnomAD Latino/admixed American	MAF–gnomAD global
rs3804100	T > C	0.047 (4.7%)	0.102 (10.2%)	0.245 (24.5%)
rs3775291	C > T	0.007 (0.7%)	0.005 (0.5%)	0.011 (1.1%)
rs3775290	C > T	0.250 (25.0%)	0.284 (28.4%)	0.327 (32.7%)

### 3.4 Main findings of the article

The results demonstrated that the analyzed SNPs (rs3804100 in *TLR2* and rs3775290 and rs3775291 in *TLR3*) presented specific allele frequencies in the studied cohort, with the most frequent minority variant being the T allele of rs3775290 (MAF = 25%) and the variants rs3775291 and rs3804100 presenting lower frequencies (MAF <5%). Analysis of the haplotypes derived from these SNPs showed a predominance of the TCC reference haplotype (∼70%), with other haplotypes distributed at lower frequencies.

No statistically significant association was found between these haplotypes/alleles and the presence or absence of COVID-19 symptoms (*p* > 0.05), either between the asymptomatic and symptomatic groups or between the different degrees of severity assessed through CCT (with lung involvement). The permutation test confirmed the absence of a significant association between the variants and the phenotypic variability of symptoms.

The results of the *in silico* comparative analysis of allele frequencies reveal a distinct genetic profile in the Belém cohort compared to reference population databases. The frequency of the minority C allele of the *TLR2* rs3804100 SNP in our sample (4.7%) was drastically lower than the frequency reported for the global population in gnomAD (24.5%). More importantly, even when compared to the most genetically similar group—the Latino/admixed American cluster (10.2%)—the frequency in Belém remains less than half. This pattern is repeated, less pronounced, but still evident, for the *TLR3* rs3775290 SNP, whose T allele has a frequency of 25.0% in Belém, compared to 28.4% in Latinos and 32.7% globally. SNP rs3775291, already rare globally (1.1%), was even rarer in the Belém sample (0.7%).

## 4 Discussion

COVID-19 continues to be a public health problem with numerous endemic foci around the world, and variants of concern (VOCs) continue to emerge in the face of parasite–host interactions and high morbidity in the elderly and people with comorbidities (Cui et al., 2025). Immunogenetic studies with haplotypes, such as this study, are important for revealing genetic patterns of ethnicities (populations), along with epigenetic and/or environmental aspects, and associating them with phenotypes/clinical–epidemiological conditions such as COVID-19 (Contes and Liu, 2025). Candidate genes, especially those encoding immune receptors that are essential in host defense, are preponderant in determining associative genetic biomarkers. In this sense, *TLR* genes (such as *TLR2* and *TLR3*) are potential typical elements for consideration in immunogenetic analyses (Bochud et al., 2007; CHEN et al., 2021).

Previous studies have demonstrated associations between *TLR2* and *TLR3* polymorphisms and COVID-19 severity and susceptibility, underscoring the importance of these receptors in viral recognition and immune activation (Alseoudy et al., 2022; Alhabibi et al., 2023). Considering that both tuberculosis and COVID-19 affect the respiratory system and that *TLR2* is crucial in IFN-γ-mediated cellular immunity, the analysis of SNP rs3804100 is relevant to understanding the heterogeneity of the host’s immune response to SARS-CoV-2 infection in terms of more frequent symptoms typical of lung involvement (Bafica et al., 2005).

Our work extends these insights by focusing on haplotype analyses within a Brazilian cohort, providing valuable data relevant to the genetic diversity and epidemiology of COVID-19 in this Amazon region (Secolin et al., 2021). The investigation of the *TLR2*–*TLR3* haplotype that includes these three SNPs may help identify other genetic variations that influence susceptibility and clinical progression of COVID-19, aiding in the development of personalized containment and treatment strategies for the disease. Thus, the study of the haplotype of SNP rs3804100 in the *TLR2* gene and SNP rs3775291 in the *TLR3* gene represents a promising approach to unravel the immunogenetic mechanisms underlying COVID-19 and its clinical manifestations.

Given *TLR2*’s critical role in the innate immune response to viral infections, especially SARS-CoV-2, the haplotype including SNP rs3804100 in the *TLR2* gene is a key topic of investigation in COVID-19 immunogenetics. The *TLR2* gene activates the release of pro-inflammatory cytokines, including TNF-α and interferon-gamma, to defend against respiratory infections (Bafica et al., 2005; Bochud et al., 2007; Khanmohammadi and Rezaei, 2021).

SNP rs3804100, located in the exon 3 of the *TLR2* gene, characterized by the substitution of thymine (T) with cytosine (C), may alter the stability of the *TLR2* protein; however, studies conducted among healthcare professionals during the first wave of the pandemic indicated that this variant is benign and showed no significant association with the severity or symptoms of COVID-19 in that population (Cheng et al., 2015; Silva et al., 2023b). In a systematic review study by Schurz et al. (2015), more severe tuberculosis cases were associated in all genetic predictive models, with a higher risk of susceptibility due to the presence of the mutant allele (C) of the TLR2 rs3804100 SNP in Asian populations (Schurz et al., 2015).

*TLR3* recognizes double-stranded viral RNA (dsRNA), activating signaling pathways that lead to the production of interferons and other antiviral cytokines, which are essential for controlling viral replication and modulating the inflammatory response (Bortolotti et al., 2021). SNP rs3775290, located in the exon 4 of the *TLR3* gene, is a genetic variant that has been studied for its influence on susceptibility to COVID-19 due to the crucial role of TLR3 in the innate immune response against viruses, including SARS-CoV-2. *TLR3* recognizes viral RNA and activates signaling pathways that promote the production of interferons and other antiviral cytokines, which are essential for controlling the infection. Studies have investigated the association of rs3775290 with the severity and susceptibility to COVID-19, seeking to understand how this variant may alter the efficiency of the host’s antiviral response. In particular, Alseoudy et al. (2022) found that the T allele of rs3775290 was associated with a higher risk of severe COVID-19 pneumonia (Alseoudy et al., 2022; Silva et al., 2023c).

The rs3775291 variant (located on exon 4 of the *TLR3* gene) is a nucleotide substitution (C>T) that can cause structural and functional changes in the TLR3 protein, potentially impacting the effectiveness of the antiviral immune response. It results in a proline-to-serine change (P554S) and has also been associated with diminished *TLR3* signaling and IFN-β production (Bucciol et al., 2022). Studies have investigated the association of this variation with COVID-19 susceptibility and severity; however, the results are inconsistent. Some studies found no significant association between SNP rs3775291 and COVID-19 severity or symptoms in specific populations (Dhangadamajhi and Rout, 2021; Silva et al., 2023c). However, research by Silva et al. (2023b) indicated that this variant may be associated with an increased risk of viral infections in Asian and American populations, suggesting a potential high-risk genetic pattern for COVID-19 in these groups (Xie and Zhu, 2020; Dhangadamajhi and Rout, 2021; Silva et al., 2023c; Silva et al., 2023c).

SNP rs3804100 was chosen because it (T > C) has already been widely studied in other respiratory diseases, such as tuberculosis, which, like COVID-19, affects the lungs (Flores-González et al., 2024). This suggests that variations in this SNP may influence the immune response in lung infections, causing instability in the formation of the TLR2 protein. More directly, Flores-González et al. (2024) demonstrated that the rs3804100 variant induces a distinct profile of *TLR2* expression and cytokine release (e.g., IL-6 and TNF-α) in response to the SARS-CoV-2 spike protein, providing a direct functional link between this SNP and the COVID-19 immune response (Flores-González et al., 2024). The rs3775291 SNP has already been associated with susceptibility to various viral infections, and variations in this SNP in a non-synonymous way, that is, that alter the structure of the TLR3 protein, can impact its immune function and, consequently, the host’s response to COVID-19 infection (Andrade et al., 2024). In the case of SNP rs3775290, this biomarker was chosen for analysis in this study because of its location in the *TLR3* gene, which encodes a key receptor for detecting RNA viruses and activating the innate immune response (Wang et al., 2015).

The *TLR2* and *TLR3* genes encode receptors essential for recognizing pathogen molecular patterns and activating essential innate immune responses. The rs3804100 variant in *TLR2* has been linked to alterations in protein stability and modulation of inflammatory activation, whereas the rs3775290 and rs3775291 variants in *TLR3* may influence viral RNA detection and subsequent interferon production (Dos Santos et al., 2025). The lack of a significant association suggests that in this group of professionals exposed and infected during the first wave of the pandemic in the Amazon region, these *TLR2* and *TLR3* variants do not play a determining role in modulating susceptibility or clinical severity of COVID-19. This may indicate that other genetic, epigenetic, environmental, or immunological factors have a greater influence on the host response, or that the genetic heterogeneity of the Amazonian population confers different risk patterns (Evdokimov et al., 2021; Minashkin et al., 2022).

The low LD observed between the SNPs evaluated (r^2^ < 0.08) indicates that these variants segregate virtually independently, which may explain the absence of strong haplotypic blocks in the study population. This genetic independence confirms that the combined effect of SNPs on the same chromosome 4 is limited, reinforcing the lack of a clearly defined risk haplotype associated with COVID-19 in the group evaluated (Salamaikina et al., 2023; Teräsjärvi et al., 2024).

From a clinical perspective, the lack of association between the tested SNPs and the severity or symptoms of COVID-19 in healthcare workers may reflect local genetic and environmental particularities. Previous studies have indicated that the immune response to viral infections is multifactorial, involving multiple genes, cellular pathways, and external influences beyond SNPs (Feng et al., 2022).

The comparison of the genetic diversity in our present study provides crucial context, showing that the allele frequencies in our Brazilian Amazonian cohort are significantly different from those observed in European populations but are more closely aligned, although still distinct, with those of other Latino/admixed American populations. This genetic distinctiveness strongly supports our conclusion that population-specific genetic architecture is a key factor in explaining the lack of association found in our study, which might be present in other, more well-studied populations. In addition, this comparative analysis underscores the unique genetic background of the Amazonian population studied and reinforces that the lack of association observed may be attributable to population-specific genetic patterns, limiting the generalizability of findings from other ethnic groups (Rodrigues de Moura et al., 2015; Silva et al., 2023c; Silva et al., 2023b).

This disparity has two critical implications, which provide a possible fundamental explanation for this study’s negative findings. First, it validates the unique genetic background of the Amazonian population studied, which is not adequately represented even in large databases that include Latinos (Lins et al., 2010). Second, and more crucial for the interpretation of our null results, it demonstrates that potentially risky or protective alleles, previously associated with COVID-19 in European and Asian populations where they are more common, are simply very rare or absent in this specific cohort. Consequently, the statistical power to detect any significant effect of these specific variants was inevitably low. Therefore, the lack of association does not necessarily invalidate findings from other studies but rather highlights that the genetic determinants of COVID-19 are highly dependent on the population context. Our negative findings are, in fact, a positive finding that highlights the importance of conducting genetic studies in diverse and underrepresented populations, as risk factors are not universal (Pena et al., 2020).

Despite the lack of statistical association, the present study contributes to mapping genetic variations and limitations of the role of these SNPs in the analyzed population. The findings reinforce the complexity of the COVID-19 genetic profile and the importance of studying diverse populations, especially those in the Amazon, whose genetic variability and environmental factors are poorly represented in large genomic databases. The lack of association between the analyzed SNPs highlights the need to address a broader panel of immunological variants and molecular mechanisms to understand how clinical response is modulated in COVID-19. These data are useful for directing future research, indicating that studies with larger sample sizes and integrative analyses involving genomics, transcriptomics, and function are necessary to elucidate relevant immunogenetic mechanisms in the Amazon population (Secolin et al., 2021).

This present study has some important limitations that should be considered when interpreting its results. A central limitation is the relatively small sample size (N = 212) and the use of a convenience sampling methodology, focused on healthcare professionals from a specific region (Belém, Pará, in the Brazilian Amazon Region). Although the calculated sample power (p = 0.94) is adequate for more common variants, this limited sample substantially reduces the statistical power to detect significant associations with low allele frequency variants (MAF <5%), such as SNPs rs3775291 and rs3804100. Consequently, the results for these rarer polymorphisms should be interpreted with extreme caution as the absence of a statistical association may reflect an inability to detect a true effect (type II error) rather than definitive evidence of its absence.

Another important consideration relates to the definition of COVID-19 cases during the early stages of the pandemic in Brazil. At that time, the criteria established by the Ministry of Health were still evolving and relied predominantly on clinical–epidemiological suspicion, with limited access to confirmatory laboratory testing (Hakim et al., 2021; Rodrigues et al., 2024). This context increased the probability of misclassification bias, with some true cases being missed (false negatives) and other respiratory infections being incorrectly categorized as COVID-19 (false positives). Additionally, the evolving understanding of COVID-19 symptomatology—ranging from asymptomatic to atypical systemic manifestations—further complicated the consistency of case definition. The lack of detailed longitudinal data also limits the analysis of the genetic influence on post-COVID-19 syndrome. Therefore, the findings should be considered hypothesis-generating for this unique population, highlighting the need for future studies with larger sample sizes, more comprehensive genetic panels, and a design that allows for more robust control of environmental and epigenetic variables (Sharpe, 2015).

The understanding of individual variations and associated genetic patterns in genomic blocks can aid in characterizing the immune response to SARS-CoV-2 and may, in the future, help in developing personalized treatment and prevention strategies for the disease (Caratachea, 2007; Aguiar et al., 2021). However, further investigations are necessary to clarify their exact role in the pathogenesis and clinical evolution of COVID-19.

## 5 Conclusion

Therefore, the *TLR2–TLR3* haplotype (SNPs rs3804100, rs3775290, and rs3775291) showed little determination in the clinic of individuals with COVID-19 in Belém (Northern Brazil), which may indicate differences in collective genetic patterns and/or epigenetic influences compared to those in other more affected populations that have the same haplotype pattern worldwide. Moreover, the results obtained in this epidemiological study provide important insights into the Brazilian Amazon population genomics about COVID-19 involvement; however, the complexity of the factors involved and the identified methodological limitations indicate the need for additional studies to strengthen the prediction and confirmation of the data presented here.

Then, it is recommended to conduct complementary research, preferably with more robust analytical designs, greater control of confounding variables, and representative samples, to validate the raised hypotheses and improve the understanding of the causal mechanisms involved. In this way, it will be possible to increase the precision of the estimates and the validity of the inferences, contributing to the development of more effective preventive strategies and interventions in the studied population.

## Data Availability

The original contributions presented in the study are included in the article/Supplementary Material; further inquiries can be directed to the corresponding authors.
